# Lipid-Lowering and Hepatoprotective Effects of Basil-Enriched Soybean Oil (BEO) in High-Fat-Diet-Fed Mice

**DOI:** 10.3390/metabo16020115

**Published:** 2026-02-05

**Authors:** Amani Tayebi, Mohammadine Moumou, Abdelhay Addous, Oussama Khibech, Niama Hammani, Youssra Salhi, Dragan Milenkovic, Ahmed Karim, Mohammed Choukri, Souliman Amrani, Hicham Harnafi

**Affiliations:** 1 Laboratory of Bioresources, Biotechnologies, Ethnopharmacology and Health, Faculty of Sciences, University Mohamed I, Oujda 60000, Morocco; amani.tayebi@ump.ac.ma (A.T.); mohammadine.moumou@ump.ac.ma (M.M.); abdelhay.addous.d23@ump.ac.ma (A.A.); niama.hammani@ump.ac.ma (N.H.); youssra.salhi@ump.ac.ma (Y.S.); a.karim@ump.ac.ma (A.K.); s.amrani@ump.ac.ma (S.A.); 2 Laboratory of Applied Chemistry and Environment (LCAE), Faculty of Sciences, University Mohamed I, Oujda 60000, Morocco; oussama.khibech.d24@ump.ac.ma; 3 Plants for Human Health Institute, Department of Food, Bioprocessing and Nutrition Sciences, North Carolina State University, Kannapolis, NC 28081, USA; dmilenk@ncsu.edu; 4 Biochemistry Laboratory, Central Laboratory Service, University Hospital Center, Oujda 60000, Morocco

**Keywords:** *Ocimum basilicum* L., antioxidants, refined soybean oil, hypolipidemic, lipid metabolism

## Abstract

**Background:** This study investigated the hypolipidemic and hepatoprotective effects of refined soybean oil supplemented with an *Ocimum basilicum* L. extract, characterized by HPLC and found to be rich in caftaric, caffeic, chicoric, and rosmarinic acids. **Methods:** After a 12-week model of diet-induced hyperlipidemia, we examined the plasma levels of TC, TG, Glucose, HDL-C, and LDL-C and the LDL-C/HDL-C ratio using enzymatic kits. The Plasma Hepatic and Biliary Marker Analysis was analysed following standardized hospital protocols with quality-controlled instrumentation. **Results:** The supplementation with Basil-Enriched Oil (BEO) resulted in a notable redistribution of lipids, significantly reducing the plasma total cholesterol (−75%), triglycerides (−96%), and glucose (−22%), while enhancing their hepatic sequestration. This was accompanied by a marked improvement in the LDL-C/HDL-C ratio and a reduction in hepatic oxidative stress (measured by MDA). Importantly, BEO preserved liver structure and prevented steatosis, despite inducing an increase in adaptive hepatomegaly. **Conclusions:** The results reveal a dual mechanism whereby the antioxidant properties of BEO collaborate with reprogrammed lipid metabolism, promoting safe hepatic storage rather than harmful circulating levels. These findings strongly advocate for the extract’s potential as a nutraceutical for addressing hyperlipidemia and related metabolic disorders by targeting both oxidative stress and lipid imbalance. Further research is required to confirm these effects in clinical settings and to confirm its long-term efficacy.

## 1. Introduction

According to the World Health Organization, cardiovascular diseases represent the major cause of mortality worldwide [1]. One of the major public health problems is metabolic disorders, particularly hyperlipidemia and associated liver damage [2]. Elevated triglycerides (TG) are increasingly recognized as a major contributor to cardiovascular risk, largely through the accumulation of atherogenic remnant lipoproteins that promote endothelial dysfunction and inflammation [3,4]. Disturbances in cholesterol distribution, particularly an unfavorable LDL-C/HDL-C profile, further amplify this effect. Current evidence suggests that the atherogenic potential relates less to total cholesterol alone and more to lipoprotein quality, oxidative modification, and the metabolic–inflammatory context in which they circulate [5,6,7]. At the same time, metabolic overload in the liver can be led by hyperlipidemia, which is responsible for increased oxidative stress and liver damage, reflected by an increase in plasma enzyme markers (ALT, AST, ALP, GGT, and LDH) as well as biliary abnormalities (total and direct bilirubin) [8].

The imbalance between the production of reactive oxygen species (ROS) and endogenous antioxidant defenses creates a state of oxidative stress, which plays a central role in initiating and progressing liver injury. The accumulation of ROS promotes lipid peroxidation, measured by an increase in malondialdehyde (MDA), and leads to structural and functional damage of cell membranes [9]. This process strongly correlates with developing hepatic steatosis, persistent inflammatory responses, and fibrosis [10].

Beyond the effectiveness of established treatments, research is progressively concentrating on the potential of natural alternatives for sustainable health management. The search for natural molecules with hypolipidemic, hepatoprotective, and antioxidant activities represents a promising approach. Sweet basil (*Ocimum basilicum* L.) is known for its richness in phenolic acids, flavonoids, and other bioactive secondary metabolites, which are traditionally used in food medicine due to their antioxidant, anti-inflammatory, and lipid-lowering properties [11,12,13]. The richness of this plant in phenolic compounds, including especially caftaric acid, caffeic acid, chicoric acid, and rosmarinic acid, explains its therapeutic potential [14,15]. These molecules exhibit synergistic antioxidant properties through several mechanisms: free-radical scavenging, chelation of pro-oxidant metals (particularly iron), and stimulation of endogenous antioxidant enzyme systems [11,12,13].

Despite evidence for the cholesterol-lowering capacities and antioxidant activity of basil extracts [16,17,18], the aqueous extract has been hardly examined in an experimental model of induced hyperlipidemia that integrates plasma biochemical, enzymatic, tissue, and histopathological analyses. In this context, our study aims to evaluate the hypolipidemic and hepatoprotective properties of an aqueous extract obtained from the residual mark after the hydrodistillation of *Ocimum basilicum*, in hyperlipidemic mice. We adopted an integrated approach, including measurement of plasma lipid parameters (TC, TG, glucose, LDL-C, and HDL-C); evaluation of liver enzymes and biliary markers including alanine transaminase (ALT), aspartate transaminase (AST), alkaline phosphatase (ALP), gamma glutamyl transferase (GGT), lactate dehydrogenase (LDH), total bilirubin (TBIL), and direct bilirubin (DBIL); quantification of lipids, proteins, and MDA in liver homogenate; and histopathological analysis of the liver. Molecular docking was used as an in silico approach to predict the possible underlying mechanisms.

This combined approach will provide a comprehensive view of the impact of the basil-enriched oil (BEO) on lipid metabolism, oxidative stress, and liver integrity, thus helping to consider its potential application as a complementary agent in the management of dyslipidemia and the prevention of cardiometabolic complications.

## 2. Materials and Methods

### 2.1. Chemicals and Plant Material

*Ocimum Basilicum* L. (sweet basil) dried leaves were obtained from a local herbal provider. Refined sunflower oil (RSO) was obtained from a supermarket in Oujda, Morocco. Its nutritional profile is detailed below (Appendix A; Table A1). Chemicals (ABTS, linoleic acid, ferrozzine, pyrocatechol, Tween 80, CuSO_4_, FeCl_2_) and solvents were primarily sourced from Sigma-Aldrich, St. Louis, MO, USA, of analytical grade. Enzymatic kits were obtained from Biosystems S.A., Barcelona, Spain.

### 2.2. Preparation of the Basil Extract (BE)

One hundred g of dried sweet Basil leaves were first cleaned to remove any solid impurities, then sieved to eliminate dust. Subsequently, the cleaned leaves were placed in a round-bottom flask, mixed with 1500 mL of distilled water, and subjected to hydrodistillation using a Clevenger-type apparatus at 100 °C for 4 h. After hydrodistillation, the essential oil was collected, and the solid residue was filtered. The remaining aqueous extract was then concentrated by gradually evaporating the water in a ventilated oven set at 45 °C.

### 2.3. HPLC-DAD Analysis of the BE

The phenolic acid-rich extract was analyzed by HPLC, using an Agilent 1100 series chromatograph (Agilent Technologies, Waldbronn, Germany) equipped with a Diode Array Detector (DAD). Chromatographic Separation was achieved on a Hypersil ODS reverse-phase (RP18) analytical column (250 × 4 mm, particle size 5 µm) at 20 °C. A 10 µL sample was injected, and the elution was carried out at a consistent flow of 1 mL/min. The mobile phase consisted of aqueous trifluoroacetic acid (pH 2.8) (solvent A) and acetonitrile (solvent B), applied using the following gradient program: 0–1 min: 0–3% B, 1–45 min: 3–40% B, 45–55 min: 40% B, 55–56 min: 0% B. Detection was carried out at 320 nm to target phenolic compounds. Compounds were identified using a phenolics database based on their retention times and UV–visible spectra [19].

### 2.4. Infusion of the BE into Refined Sunflower Oil (RSO) and Preparation of Diets

The infusion of active compounds from the BE into the RSO was facilitated using an ultrasonic bath (Elmasonic, Singen, Germany). The mixture (1% of the BE in the RSO) was sonicated for 10 min at a controlled temperature of 30 ± 2 °C with a frequency of 50/60 Hz and an ultrasonic intensity of approximately 0.0485 W/cm^2^. The obtained enriched oil (BEO) was filtered to remove solid insoluble residues and then stored at 4 °C in a tinted glass bottle until use.

In this study, four types of diets were prepared, as outlined by Harnafi et al. [16]. The first was a standard normal diet (ND), formulated specifically for laboratory mice and supplied by the Provimac, Meknes, Morocco. The second (OD) was a standard diet (84%) supplemented with RSO (16%). The third was a hypercholesterolemic diet (OCD), composed of 82.3% standard diet, 16% RSO, 1.5% cholesterol, and 0.2% deoxycholic acid. The fourth (OCBD) was a hypercholesterolemic diet in which the RSO was replaced by the basil-enriched oil. ND had a lower caloric density than the lipid-supplemented diets, whereas OD, OCD, and OCBD were approximately isocaloric (~4.43–4.50 kcal/g). Minor caloric variations resulted from the addition of cholesterol or basil-enriched oil, but the diets were designed to maintain comparable energy content to allow for proper interpretation of lipid-induced effects. Detailed compositional values are provided in the Appendix B; Table A2.

### 2.5. Animal Study Design

Male Swiss albino mice (28–30 g; 8 weeks old) were bred and maintained in the animal facility of the Faculty of Sciences, Mohammed I University, Oujda. The animals were housed under controlled environmental conditions (22 °C, 12 h light/dark cycle) with free access to food (Appendix B; Table A2) and water. After two weeks of acclimation, the mice were randomly divided into four groups (*n* = 6), each receiving one of the four experimental diets for a period of 12 weeks. The first group was fed the standard normolipidemic diet, serving as the normal control. The second group received the standard diet added with normal RSO (OD). The third group was given the hypercholesterolemic diet (OCD), and the fourth received the hypercholesterolemic diet added with the basil-enriched oil (OCBD). The dose of basil extract (BE) consumed by mice in the OCBD group corresponded to approximately 213 mg/kg/day.

At the end of the experiment and after a 12 h fast, blood was collected via retro-orbital puncture under anesthesia. The mice were then euthanized. The liver, adipose tissue, heart, and kidneys were excised, washed in cold normal saline, and weighed. All animal experiments were conducted in compliance with the European Directive 2010/63 on the protection of animals used for scientific purposes and were approved by the institutional ethics committee for laboratory animal use (Approval No #044/2024). Every precaution was taken during handling and treatment to minimize animal discomfort and suffering.

### 2.6. Biochemical Analysis

#### 2.6.1. Plasma Lipid and Glucose Analysis

Enzymatic kits Biosystems S.A., Barcelona, Spain, were used to quantify plasma total cholesterol (TC), triglycerides (TG), LDL-C, HDL-C, and glucose. All plasma concentrations were expressed in mg/dL.

#### 2.6.2. Plasma Hepatic and Biliary Marker Analysis

The plasma key biomarkers of liver integrity were measured at the 12-week endpoint using enzyme-specific kits at the University Hospital Center of Mohammed First University (Oujda). This included the hepatic enzymes (alanine aminotransferase “C-ALT2F”, Aspartate aminotransferase “C-AST2F”, Alkaline phosphatase “C-Alkp2F”, and Lactate dehydrogenase “C-LDH”) and Biliary Function Markers (Total bilirubin “C-BilT”, Direct bilirubin “C-BilD”, and Gamma-glutamyl transferase “C-GGT2”).

C-ALT2F, C-AST2F, and C-Alkp2F were expressed in U/L. whereas bilirubin markers and GGT were expressed in mg/L. All analyses followed standardized hospital protocols with quality-controlled instrumentation.

#### 2.6.3. Hepatic Lipids Analysis

Liver lipids were extracted using a modified Haug and Hostmark method [20]. One g of tissue was homogenized in 10 mL of isopropanol, stored 24 h at 4 °C, and centrifuged at 2500 rpm for 15 min. Ten µL of supernatant was incubated with the enzymatic kits (37 °C, 10 min). Then, the absorbance was read at 500 nm. TC and TG concentrations were expressed in mg/g liver.

#### 2.6.4. Hepatic Lipid Peroxidation

Lipid peroxidation was evaluated by measuring the malondialdehyde (MDA) content, as described by Mamri et al. [21]. 0.5 mL of liver homogenate was mixed with 1 mL of 30% trichloroacetic acid, and centrifuged at 2500 rpm for 10 min. Then, 0.5 mL of the supernatant was reacted with 1 mL of 0.67% thiobarbituric acid at 90 °C for 15 min. The absorbance was measured at 532 nm to determine the concentration of MDA expressed in nmol per gram of tissue, with an extinction coefficient (ε) of 1.56 × 10^5^ M^−1^·cm^−1^.

#### 2.6.5. Liver Protein Quantification

Twenty µL of the liver homogenate supernatant, previously extracted using a phosphate buffer (0.1 M, pH 7.4), was mixed with 200 µL of Bradford reagent and incubated for ten minutes. The absorbance was measured at 595 nanometers. A bovine serum albumin (BSA) standard curve was used to determine protein concentration in milligrams per gram of tissue.

#### 2.6.6. Liver Histopathology

Liver sections were fixed in 10% buffered formalin, paraffin-embedded, sectioned, and stained with hematoxylin and eosin (H&E). Slides were examined under light microscopy. In addition, H&E-stained sections were semi-quantitatively scored using the NAFLD Activity Score (NAS), including steatosis (0–3), lobular inflammation (0–3), and hepatocellular ballooning (0–2). Scoring was performed independently by two evaluators blinded to the experimental groups; discrepancies were resolved by consensus.

### 2.7. Molecular Docking with PyRx & AutoDock Vina

**Figure A1:**
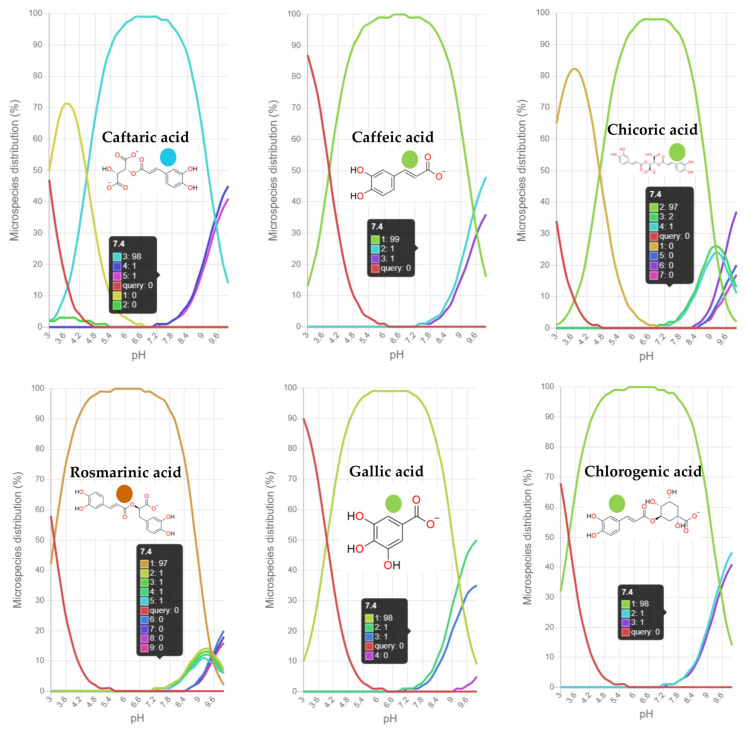
pH-dependent microspecies distribution of the six major phenolic acids identified in the basil extract (caftaric, caffeic, chicoric, rosmarinic, gallic, and chlorogenic acids). The highlighted point at pH 7.4 indicates the predominant microspecies used for docking simulations.

The six phenolic acids, namely gallic, chlorogenic, caftaric, caffeic, chicoric, and rosmarinic, were sketched in PyRx v0.9.8, converted to canonical SMILES, merged into a multi-ligand SDF, and geometry-optimized with the UFF force field; lowest-energy conformers were exported as PDBQT files with Gasteiger charges and automatically assigned rotatable bonds [22]. Ionization and microspecies distribution of each ligand as a function of pH were predicted with the SwissADME web server (Swiss Institute of Bioinformatics), and the predominant microspecies at physiological pH 7.4 (carboxylate groups deprotonated, phenolic hydroxyls neutral) was selected for docking (Appendix C; Figure A1).

Six high-resolution protein structures were selected for molecular docking based on their physiological relevance to oxidative stress, lipid metabolism, and inflammation. These included Arachidonate 15-lipoxygenase type B (ALOX15B, 7LAF), an enzyme catalyzing the lipid peroxidation involved in oxidative stress and inflammation; Farnesoid X receptor (FXR, 6A60), a nuclear receptor regulating bile acid synthesis and lipid metabolism; 5′-AMP-activated protein kinase (AMPK, 8BIK), a key cellular energy sensor promoting fatty acid oxidation and glucose homeostasis; Kelch-like ECH-associated protein 1 (KEAP1, 6TYM), which negatively regulates the Nrf2 antioxidant pathway under basal conditions but, under oxidative stress, promotes Nrf2 activation by facilitating its release and nuclear translocation; Peroxisome proliferator-activated receptor alpha (PPAR-α, 6KBA), a transcription factor controlling fatty acid catabolism and lipid homeostasis; and Sirtuin 1 (SIRT1, 4I5I), a NAD^+^-dependent histone deacetylase that regulates oxidative stress, inflammation, and metabolic pathways primarily through the deacetylation of histone residues such as H3K9Ac and H4K16Ac.

Prior to preparation in AutoDockTools v1.5.7, each protein structure was submitted to the PDB2PQR server (pH 7.4) to assign protonation states and atomic charges consistent with physiological conditions while preserving catalytic residues, structurally essential cofactors, and metal coordination. All protein structures were pre-processed in AutoDock Tools 1.5.7 by removing crystallographic waters and non-essential hetero ligands, adding polar hydrogens and Kollman charges, and retaining catalytically or structurally essential cofactors and metal ions where present. Docking was performed with AutoDock Vina 1.1.2 (exhaustiveness = 8) via the PyRx interface [23]; Vina employs an implicit-solvent scoring function with a distance-dependent dielectric to approximate ionic screening, so ionic strength was treated implicitly and docking scores were interpreted as relative binding affinities within each target rather than absolute binding free energies. Protocol validation was carried out on the two co-crystallized systems: for ALOX15B (7LAF), redocking reproduced the native pose with an RMSD of 1.528 Å, and for FXR (6A60) with an RMSD of 0.388 Å, both within the commonly accepted reliability threshold of 2 Å. Focused grids centered on the experimental binding sites were then applied for these two receptors. For the remaining four structures (AMPK 8BIK, KEAP1 6TYM, PPAR-α 6KBA, and SIRT1 4I5I), blind docking was executed over the full receptor surface. Blind docking was chosen for these multi-domain receptors because they harbor several plausible ligandable cavities, and the available co-crystal structures do not unambiguously define the relevant pocket for small, highly polar phenolic acids. Scanning the entire surface thus reduces pocket-selection bias and allows the algorithm to identify the most energetically favorable binding sites in an unbiased manner. For each ligand, the top-scoring pose was retained for subsequent 3D and 2D interaction analyses in Discovery Studio 2024 and PyMOL 2.5.

### 2.8. In Silico ADMET and Toxicity Prediction

The ADMET and toxicity profiles of the six major phenolic acids (caftaric, caffeic, chicoric, rosmarinic, gallic, and chlorogenic acids) were predicted in silico using ADMET-AI and ProTox-III [24,25,26]. Canonical SMILES of each compound were submitted to ADMET-AI under the default human oral exposure model to estimate key pharmacokinetic parameters, including human intestinal absorption, oral bioavailability, CYP2D6/CYP3A4 inhibition probabilities, hepatocellular clearance, and half-life. The same SMILES were then processed with the ProTox-III web server to obtain acute toxicity (LD_50_, toxicity class) and the predicted probabilities of hepatotoxicity, cardiotoxicity, carcinogenicity, mutagenicity, and cytotoxicity. These computational outputs were used as supportive evidence to contextualize the safety and pharmacokinetic behavior of the phenolic acids.

### 2.9. Determination of ABTS Radical Scavenging Activity of the BE

The ABTS assay, adapted from El Bouzidi et al. [27], was used to measure the free-radical-scavenging capacity of BE. A 7 mM ABTS stock solution was prepared by dissolving 19.2 mg of ABTS (2,2′-azino-bis(3-ethylbenzothiazoline-6-sulfonic acid) diammonium salt) in 5 mL of distilled water. Then, 88 µL of a 140 mM potassium persulfate (K_2_S_2_O_8_) solution was added to the ABTS solution to obtain a final concentration of 2.45 mM potassium persulfate, and the mixture was incubated in darkness (room temperature, 18 h) to generate the ABTS•^+^ radical cation. This solution was diluted with ethanol until its absorbance reached 0.70 ± 0.02 at 750 nm.

For the test, 20 µL of the sample was combined with 200 µL of the diluted ABTS•^+^ solution. After 10 min of dark incubation at room temperature, we recorded the absorbance at 734 nm. The radical-scavenging percentage was calculated using the following formula:

‘Radical scavenging activity (%) = [(A control − A sample)/A control] × 100’

‘A control’ represents the blank absorbance, and ‘A sample’ is the absorbance of the test sample.

### 2.10. Estimation of Copper Chelating Capacity of the BE

This assay was assessed using pyrocatechol violet (PV), which forms a blue Cu^2+^-PV complex detectable at 632 nm. A yellow color shift and decreased absorbance indicate that the chelating agents prevent this complex formation. We followed a modified Kähkönen et al. protocol [28].

Two-hundred fifty µL of a test solution were mixed with 1 mL of sodium acetate buffer (50 mM, pH 6.0) and 25 µL CuSO_4_ solution (5 mM). After 30 min at room temperature, 25 µL of PV solution was added. Following another 30 min incubation, absorbance was measured at 632 nm. A blank (distilled water plus extract) served as the negative control. Chelation inhibition was calculated as:

‘Inhibition (%) = [(A_0_ − A_1_)/A_0_] × 100’

‘A_0_’ is the negative control absorbance; ‘A_1_’ is the absorbance with the test sample.

### 2.11. Determination of Ferrous Ion (Fe^2+^) Chelating Activity of the BE

We determined Fe^2+^ chelation using the ferrozine method, reporting efficiency as IC_50_ (concentration for 50% chelation). Lower IC_50_ indicates stronger activity. This assay relies on the disruption of the Fe^2+^-ferrozine complex (absorbance at 562 nm) by chelators [29].

A 250 µL test sample (various concentrations) was mixed with 1 mL acetate buffer (0.1 M, pH 4.9) and 25 µL FeCl_2_ (2 mM). After 30 min of incubation, 100 µL ferrozine (5 mM) was added. After incubation for 30 min, the absorbance was read at 562 nm. Distilled water replaced the sample for the negative control. The inhibition percentage was:

‘Inhibition (%) = [(A_0_ − A_1_)/A_0_] × 100’

‘A’ is the negative control absorbance; ‘A_1_’ is the sample absorbance.

### 2.12. Inhibition of Linoleic Acid Oxidation by the BE

This test measures the inhibition of linoleic acid oxidation in an emulsion containing β-carotene as a marker [30]. We dissolved 2 mg β-carotene in 1 mL chloroform, added 20 mg linoleic acid and 200 mg Tween-80, and removed chloroform using a rotavapor (40 °C). The residue was emulsified in 100 mL of distilled water under vigorous stirring.

We added 50 µL extract to 2.45 mL of this emulsion. Absorbance at 490 nm was measured immediately (A_0_) and after 2 h at 50 °C (A_1_). A control used distilled water plus extract. Oxidized β-carotene percentage was:

‘Oxidized linoleic acid (%) = [(A_0_ − A_1_)/A_0_] × 100’

### 2.13. Statistical Analysis

All assays were performed in at least triplicate, except for in vivo experiments, where each group consisted of six mice (*n* = 6). Results are mean ± SEM. Data were first analyzed using one-way ANOVA to determine whether there were overall differences between groups. When ANOVA indicated a significant effect, Tukey’s multiple comparisons test was applied to identify which specific groups differed. Statistical significance was considered at *p* < 0.05. Data analysis and graphing were performed using GraphPad Prism (v10.2.0).

## 3. Results

### 3.1. HPLC Analysis of the BE

**Figure 1:**
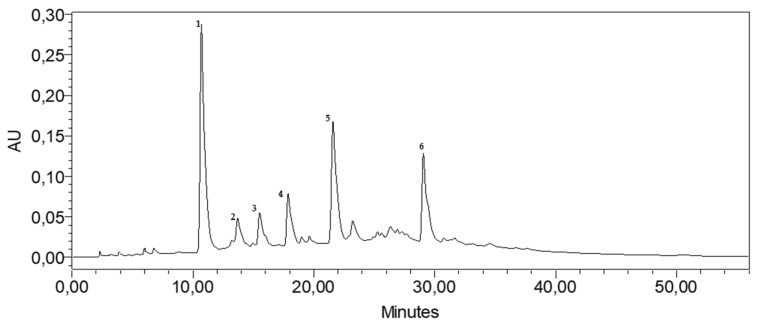
HPLC phenolic profile of BE; 1: caftaric acid; 2: gallic acid; 3: chlorogenic acid; 4: caffeic acid; 5: chicoric acid; 6: rosmarinic acid.

The HPLC chromatogram (Figure 1) revealed the presence of 19 phenolic compounds in the aqueous basil extract. The major identified compounds were caftaric acid (Peak 1), gallic acid (Peak 2), chlorogenic acid (Peak 3), caffeic acid (Peak 4), chicoric acid (Peak 5), and rosmarinic acid (Peak 6), with relative area percentages of 35.03%, 4.20%, 3.39%, 8.36%, 21.15%, and 16.33%, respectively.

### 3.2. Metabolic Effects of the Basil-Enriched Oil (BEO) in Treated Mice

#### 3.2.1. Organ Weight Modifications

**Figure 2:**
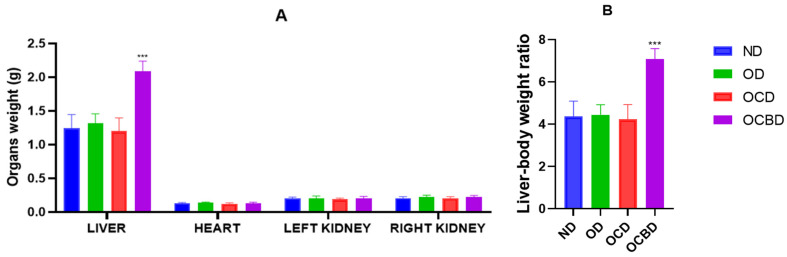
Effect of the experimental diets on mice organs’ weight (**A**) and liver–body weight ratio (**B**). Normal Diet (ND), diet rich in oil (OD), diet rich in oil and cholesterol (OCD), diet rich in cholesterol and basil-enriched oil (OCBD). Statistical significance: *** *p* < 0.001 (OCBD vs. OCD).

Figure 2 illustrates the changes in organ weights after 12 weeks of treatment. Liver mass showed the most pronounced dietary response, with the BEO-supplemented group (OCBD) displaying a 43% increase in mass compared to hyperlipidemic controls (OCD; *p* < 0.001). In contrast, liver weights in the other groups remained unchanged. Moreover, no statistically significant differences were observed in the weights of the heart, kidney, or adipose tissue among the experimental groups.

#### 3.2.2. Metabolic Parameter Distribution

**Figure 3:**
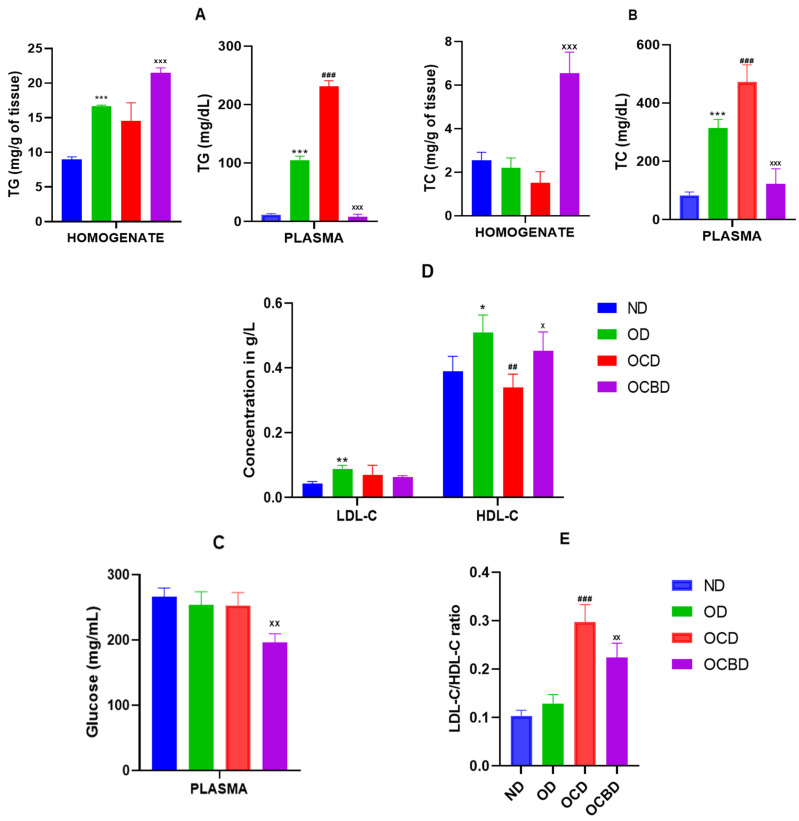
Effect of BEO on LDL-C-HDL-C (**D**), and LDL-C/HDL-C (**E**) ratio plasma levels, TG (**A**), TC (**B**), and Glucose (**C**) plasma and liver levels in 12 weeks of treatment. Normal diet (ND), diet rich in oil (OD), diet rich in oil and cholesterol (OCD), diet rich in cholesterol and basil-enriched oil (OCBD). Statistical significance: * *p* < 0.05, ** *p* < 0.01, *** *p* < 0.001 (OD vs. ND); ^##^ *p* < 0.01, ^###^ *p* < 0.001 (OCD vs. OD); ^X^ *p* < 0.05, ^XX^ *p* < 0.01, ^XXX^ *p* < 0.001 (OCBD vs. OCD).

The Triglyceride (TG) distribution revealed pronounced tissue–blood partitioning. The hepatic TG content increased 33% (*p* < 0.001) in the OCBD group against the OCD one, while circulating TG levels decreased markedly (96%) (Figure 2A). Cholesterol metabolism showed parallel divergence; plasma total cholesterol (TC) declined 75% (*p* < 0.001) in the OCBD group despite significant hepatic TC accumulation (79% above hyperlipidemic diet without enriched oil) (Figure 3B).

Lipoprotein analysis further confirmed the lipid-regulating effect of the basil extract. In hypercholesterolemic mice (OCD), both LDL-C and HDL-C were altered, with a marked reduction of HDL (−33%) and a moderate increase in the LDL/HDL ratio, indicating a disturbed lipoprotein balance. Basil supplementation (OCBD) significantly improved this profile by reducing LDL-C (−24%) and elevating HDL-C (+32%; *p* < 0.05) compared to the OCD group, leading to a 24% decrease in the LDL-C/HDL-C ratio (*p* < 0.01). In contrast, oil-rich feeding alone (OD) slightly increased both LDL-C (*p* < 0.05) and HDL-C (*p* < 0.01) relative to the normal diet (ND), maintaining a low LDL/HDL ratio (Figure 3D,E).

#### 3.2.3. Effect of the BEO on Hepatic Function and Oxidative Stress

**Figure 4:**
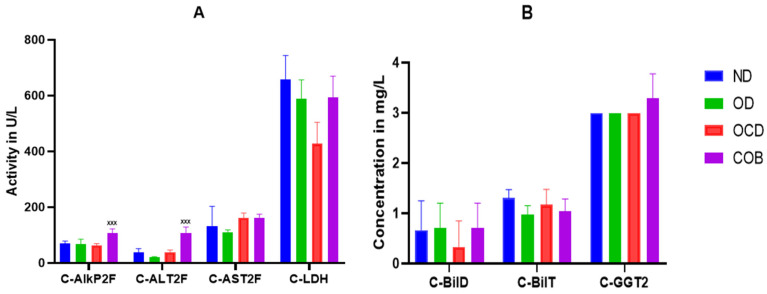
Effect of the BEO on hepatic function markers: ALT, AST, ALP, and LDH (**A**), and direct bilirubin (BILD), total bilirubin (BILT), and γ-glutamyl transferase (GGT) (**B**). Normal diet (ND), diet rich in oil (OD), diet rich in oil and cholesterol (OCD), diet rich in cholesterol and basil-enriched oil (OCBD). Statistical significance: ^XXX^ *p* < 0.001 (OCBD vs. OCD).

Plasma analysis of hepatic function markers revealed signs of selective hepatocellular stress, as evidenced by significant increases in alkaline phosphatase and alanine aminotransferase levels by 40% (*p* < 0.001) and 63% (*p* < 0.001), respectively in the OCBD group (Figure 4A), their absolute values remained within the normal physiological range for Swiss mice (ALT: 45–120 U/L), supporting the interpretation of a eustress-type adaptive response. In contrast, other hepatic and biliary parameters, including aspartate aminotrasferase, bilirubin fractions, and γ-glutamyl transferase, remained unaltered among the experimental groups.

**Figure 5:**
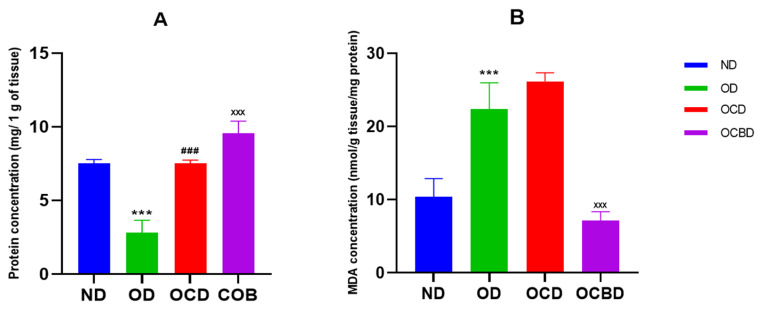
Effect of RSO/A-Bs on liver homogenate protein concentrations (**A**) and MDA concentration levels (**B**) in 12 weeks of treatment. Normal diet (ND), diet rich in oil (OD), diet rich in oil and cholesterol (OCD), diet rich in cholesterol and basil-enriched oil (OCBD). Statistical significance: *** *p* < 0.001 (OD vs. ND); ^###^ *p* < 0.001 (OCD vs. OD); ^XXX^ *p* < 0.001 (OBD vs. OCD).

Hepatic oxidative stress assessment revealed substantial protective effects of basil-enriched oil supplementation. Specifically, MDA levels were reduced by 73% in OCBD-fed mice compared to OCD-fed controls (*p* < 0.001) (Figure 5A), indicating decreased lipid peroxidation. In parallel, total hepatic protein content increased by 20% in OCBD-fed mice (Figure 5B), suggesting a potential stabilization of hepatic protein homeostasis under conditions of enhanced antioxidant protection.

#### 3.2.4. Histopathological Correlates of Dietary Interventions

**Figure 6:**
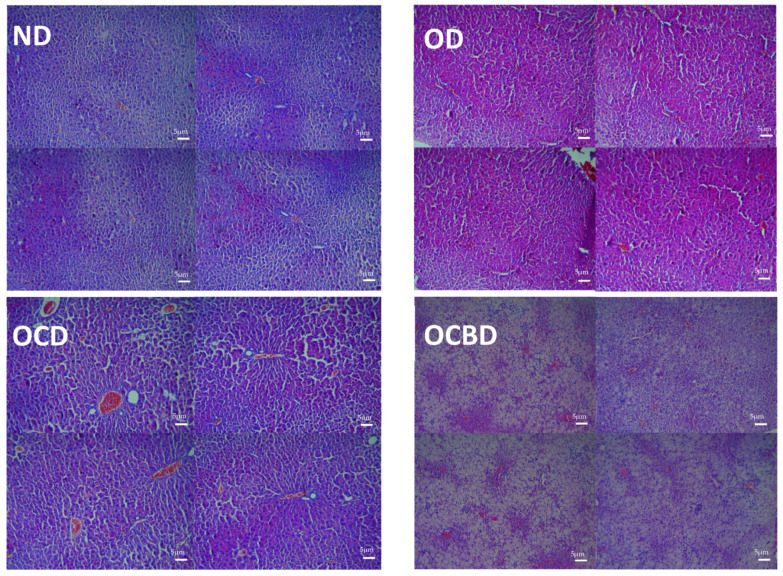
Representative H&E-stained liver sections from mice receiving different experimental diets: normal diet (ND), diet rich in oil (OD), diet rich in oil and cholesterol (OCD), diet rich in cholesterol and basil-enriched oil (OCBD). Each panel contains images of four distinct animals of the same group. Sections illustrate overall lobular architecture and key features scored using the NAFLD Activity Score (NAS): steatosis, lobular inflammation, and hepatocellular ballooning. Scale bars = 5 µm.

Microscopic examination revealed graded hepatic responses to dietary regimens (Figure 6). Normal-diet animals maintained intact lobular architecture without pathological changes, while oil-enriched diets induced early metabolic stress, manifesting as mild cytoplasmic vacuolization and sinusoidal dilation. The cholesterol-supplemented groups developed severe microvesicular steatosis with extensive vacuolization, significant sinusoidal distension, and advanced parenchymal disruption. However, basil co-administration substantially attenuated these pathologies, demonstrating residual lipid vacuoles and localized inflammatory infiltrates but markedly preserving tissue architecture with only focal sinusoidal alterations. To further standardize these observations, NAS sub-scores (steatosis, lobular inflammation, and ballooning) and total NAS were determined on H&E sections under blinded conditions and are reported in Table 1

### 3.3. Molecular Docking of BE

As reported in Table 2, caffeoyl diesters are the prevailing scaffold across targets, with docking scores mirroring the bioassay trends. Chicoric acid ranks first overall (SIRT1, −10.5 kcal·mol^−1^; FXR, −9.4; KEAP1, −9.7), consistent with cytoprotective deacetylation (SIRT1), lipid-regulatory signaling (FXR), and KEAP1-NRF2 activation, aligning with the anti-stress/anti-inflammatory readouts [5]. Rosmarinic acid performs consistently well (ALOX15B, −8.5; AMPK, −7.7; KEAP1, −9.5; PPAR-α, −8.5), accounting for reduced β-carotene/linoleate peroxidation and strong ABTS scavenging likely aided by Fe^2+^-biased chelation via KEAP1-NRF2; PPAR-α/FXR engagement further supports lipid homeostasis and dampened inflammation. Chlorogenic acid contributes at KEAP1 (−9.3) and PPAR-α (−8.4), while caftaric (SIRT1, −9.2; KEAP1, −8.3) and caffeic (KEAP1, −7.1) acids provide smaller yet meaningful effects.

**Figure 7:**
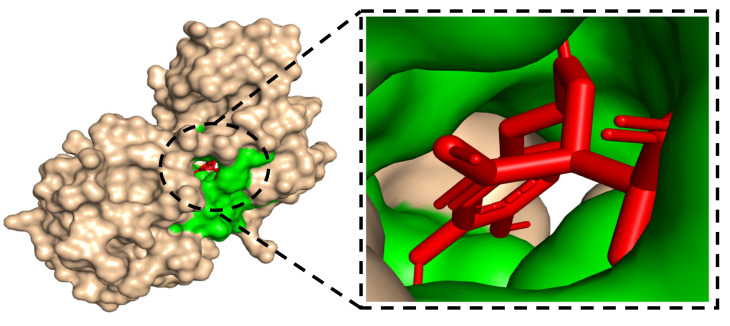
SIRT1-chicoric acid complex in PyMOL: binding pocket shown in green and docked ligand in red.

Figure 7 provides a 3D PyMOL visualization of the docked chicoric acid within the SIRT1 binding pocket (pocket highlighted in green and ligand in red), confirming a well-accommodated pose inside the cavity.

**Figure 8:**
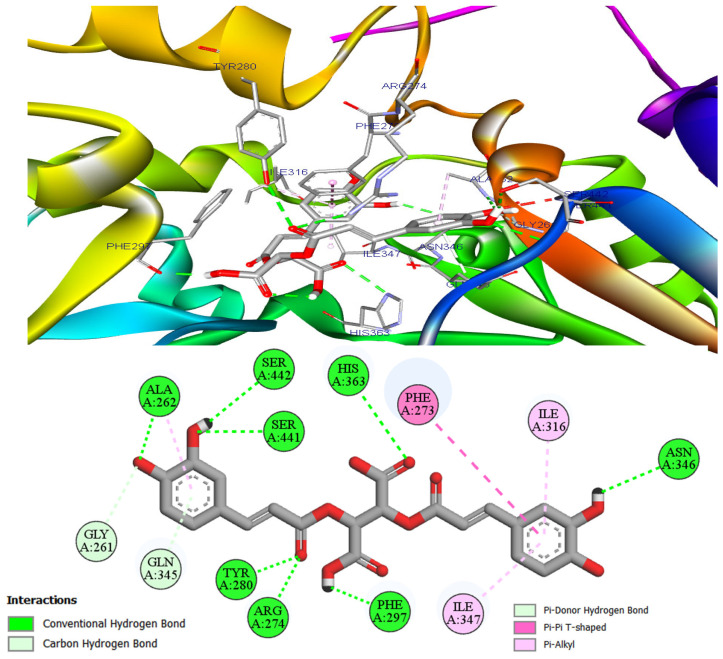
A 3D and a 2D visualization of the SIRT1-chicoric acid complex, highlighting the binding pocket and ligand pose.

Figure 8 shows that chicoric acid is stabilized in the SIRT1 pocket by an extensive hydrogen-bonding network involving its carbonyl and phenolic OH groups. Additional polar contacts include a C–H···O interaction with Glu261 and a π–donor hydrogen bond with Gln345. Hydrophobic stabilization is provided by π–alkyl contacts with Ile316, Ile347, and Ala262, while Phe273 forms a T-shaped π–π interaction that locks one caffeoyl ring in a cross-stacked geometry.

### 3.4. ADMET Analysis

**Figure 9:**
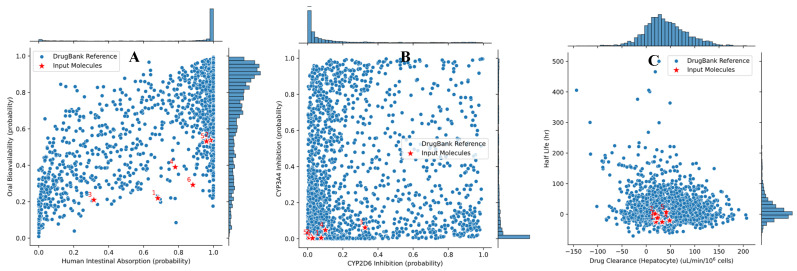
ADMET-AI profile of the six major phenolic acids identified in the basil-enriched oil compared with DrugBank reference drugs. (**A**) Human intestinal absorption vs. oral bioavailability probabilities. (**B**) Predicted CYP2D6 vs. CYP3A4 inhibition probabilities. (**C**) Predicted hepatic drug clearance vs. half-life probability. Blue dots represent DrugBank reference compounds; red stars represent the six input molecules, numbered as follows: 1, caftaric acid; 2, caffeic acid; 3, chicoric acid; 4, rosmarinic acid; 5, gallic acid; 6, chlorogenic acid.

As shown in Figure 9, the six major phenolic acids (1, caftaric acid; 2, caffeic acid; 3, chicoric acid; 4, rosmarinic acid; 5, gallic acid; 6, chlorogenic acid) occupy a non-alarming region of the ADMET-AI chemical space when overlaid on DrugBank reference compounds. Panel A indicates that these molecules combine good predicted human intestinal absorption with low-to-moderate oral bioavailability, a pattern typical of polar, polyhydroxylated natural products that are nevertheless able to reach the systemic circulation. In panel B, all six stars cluster in the lower-left corner of the plot, with very low probabilities of CYP2D6 and CYP3A4 inhibition, suggesting a limited potential for major cytochrome-mediated drug–drug interactions. Panel C shows that the compounds cluster in a region of moderate predicted hepatocellular clearance and short-to-intermediate half-lives, a combination compatible with sufficient metabolic turnover to avoid accumulation while still allowing meaningful systemic exposure. Complementarily, the ProTox-III predictions summarized in Table 3 point to a favorable toxicological profile: all phenolic acids display relatively high LD_50_ values (2000–5000 mg/kg), corresponding to toxicity classes 4–5, and are uniformly predicted as inactive toward hepatotoxicity, cardiotoxicity, mutagenicity, and cytotoxicity, with high associated probabilities. Only caffeic and gallic acids show a possible carcinogenic signal, and even in these cases, the probability is moderate and remains compatible with their wide use as dietary polyphenols.

### 3.5. Antioxidant Activity of the BE

The assessment of the antioxidant properties of the aqueous extract of the BE demonstrated strong radical-neutralizing and metal ion-chelating activities, as detailed in Table 4. Thus, the extract scavenges the ABTS radicals with an IC_50_ value of 17.65 ± 0.10 μg/mL, indicating its possible capacity to neutralize free radicals leading to oxidative stress. Furthermore, the BE shows a preventive effect against linoleic acid oxidation, with an IC_50_ of 111.31 ± 0.01 μg/mL, which could support its beneficial effect at the cell level.

The extract also demonstrated ferrous and copper ions chelation capabilities, reflected in IC_50_ values of 153.80 ± 2.50 μg/mL. and 0.544 ± 0.009 mg/m, respectively.

## 4. Discussion

This study shows that the BE exhibits a strong in vitro antioxidant profile, alongside significant in vivo metabolic and hepatic effects in a mouse model of hyperlipidemia induced by a hypercholesterolemic diet.

The extract operates through two complementary antioxidant mechanisms, electron/hydrogen-transfer scavenging and transition-metal regulation. The robust quenching of ABTS•^+^ indicates an efficient capacity for both single-electron and hydrogen-atom transfer, while the β-carotene/linoleate assay demonstrates its ability to break the lipid peroxidation chain [28,29,30]. The extract displays a notable capacity to chelate Fe^2+^ and Cu^2+^, likely due to its catechol and hydroxyl-rich phenolic compounds, which can mitigate Fenton/Haber–Weiss reactions and suppress upstream hydroxyl–radical propagation before radical capture [17,27]. Minor constituents in the extract may also contribute to this metal-binding activity.

Other polyphenol-rich extracts, such as those from rosemary, grape seed, and green tea, have been widely reported to possess strong antioxidant and radical-scavenging activities [31,32,33,34]. This supports that plant-derived phenolic acids, including those identified in our basil extract, consistently contribute to protection against oxidative stress.

The HPLC analysis reveals the presence of caftaric, caffeic, chicoric, rosmarinic, gallic, and chlorogenic acids that could contribute both to radical scavenging and metal binding [14,15]. Caffeic and caftaric acids were demonstrated to provide strong hydrogen donation [15,29], and chicoric acid can enhance Fe^2+^ affinity [12]. These attributes collectively suggest a reduced initiation and propagation of lipid oxidation in biological and food-mimetic systems.

In the mice hypercholesterolemic model, adding basil extract to the oil is consistent with a redistribution of lipid fluxes away from plasma and toward the liver, suggesting hepatocellular uptake and packaging into lipid droplets as a protective buffer against circulating lipotoxic which refer to excess free fatty acids and lipid intermediates that can induce cellular stress, oxidative damage, and inflammation [11,12,16,35,36,37,38]. While BEO supplementation demonstrated antioxidant and lipid-regulating properties, classical markers of liver injury were not present in this model. Hence, BEO’s hepatoprotective effects should be interpreted as supporting hepatic adaptation under metabolic stress, rather than protection against pre-existing liver damage. Conceptually, this can arise from coordinated changes in uptake (e.g., SR-B1 [39], LDL-R with reduced PCSK9-mediated degradation, FAT/CD36/FATP5 (SLC27A5), ACSL1/ACSL5 for fatty-acid activation), lipoprotein assembly/export (MTP/ApoB-dependent VLDL, modulation of hepatic lipase LIPC and peripheral LPL for remodeling) [40], and lipid-droplet biogenesis (DGAT1/2; perilipins, PLIN2/PLIN5, GPAT/AGPAT steps), such that the liver temporarily sequesters dietary lipids in a less atherogenic compartment while maintaining controlled lipolysis via ATGL (PNPLA2)/HSL (LIPE) and lipophagy (ATG5/ATG7, TFEB) [41].

The enzyme pattern ALT prominence with near-stable AST, moderate ALP/GGT dynamics, and overall tempered bilirubin signals fits an active, adaptive remodeling rather than frank cytolysis or cholestatic failure [9,11,12,35,36,37]. Cytosolic ALT sensitivity to membrane perturbation can rise during high metabolic throughput, while unchanged AST (with a substantial mitochondrial pool) argues against widespread mitochondrial necrosis [9].

Importantly, when examining the absolute enzymatic values, ALT in OCBD mice (106.71 ± 8.43 U/L) remained within the upper physiological range typically reported for Swiss mice (≈45–120 U/L) [42,43]. Although ALT increased by 63%, it remains within normal limits, consistent with adaptive metabolic remodeling rather than hepatocellular injury [44,45]. AST (162.5 ± 4.67 U/L) and bilirubin fractions showed no significant deviation from the controls, γ-glutamyl transferase and other cholestatic markers remained stable, and LDH activity, though slightly elevated in OCBD mice (592.66 ± 29.09 U/L), did not reach levels indicative of overt hepatocellular necrosis.

Taken together, these biochemical features support a eustress-type hepatocellular adaptation to accelerated lipid processing rather than broad hepatic injury or cholestasis. The ALT-dominant pattern, stable AST, and modest ALP/GGT dynamics are consistent with cytosolic membrane perturbation during heightened metabolic throughput, while the absence of significant changes in AST (which has a larger mitochondrial pool) argues against mitochondrial necrosis. Likewise, the mild shifts in canalicular markers likely reflect membrane remodeling and bile flow adjustments in response to altered lipid export rather than true biliary obstruction [9]. This adaptive interpretation is reinforced by preserved lobular architecture on histology and by the reduction in hepatic MDA levels, indicating controlled oxidative stress and a protective hepatic response to the basil-enriched oil.

Marked attenuation of lipid peroxidation is congruent with the phenolic-driven antioxidant milieu documented in vitro [16,28,29,30,38]. A lower peroxidative tone preserves mitochondrial function, supporting β-oxidation and preventing the feed-forward loop where ROS and dysfunctional organelles exacerbate steatosis and cell death [46,47,48,49,50,51]. Histology that preserves lobular architecture with reduced steatosis/necrosis is precisely what one expects when lipid uptake/packaging is coupled to controlled redox status rather than left to unchecked oxidative injury [11,12,35,36,37].

The observed pattern is compatible with this “buffering” model and with improved glycemic homeostasis, in which polyphenols interact with multiple metabolic and antioxidant regulators to optimize substrate handling and hepatic defense. AMPK activation phosphorylates ACC (ACACA), lowering malonyl-CoA and disinhibiting CPT1A-driven β-oxidation, while SIRT1 deacetylation of PGC-1α enhances mitochondrial fatty acid oxidation (CPT1A, ACOX1, ACADM) [52]. Concurrently, PPAR-α/PGC-1α activation [53] and FXR–SHP signaling repress lipogenesis via SREBP-1c/ChREBP (↓FASN, ↓SCD1), and cholesterol handling improves through ABCA1/ABCG1-mediated efflux and bile acid conversion (CYP7A1 under LXR/FXR–SHP feedback), contributing to a lower LDL-C/HDL-C ratio. KEAP1/NRF2 activation induces antioxidant genes (↑NQO1, ↑HO-1, ↑GCLC) [46], while dampened TLR4→NF-κB/NLRP3 signaling reduces inflammatory tone and lipid peroxidation, consistent with the preserved lobular architecture and reduced hepatic MDA observed [54,55]. Together, these coordinated pathways suggest that basil phenolics simultaneously support lipid homeostasis, mitochondrial competence, redox balance, and anti-inflammatory responses, forming a mechanistic bridge between the in vivo antioxidant and hepatoprotective effects. To further refine the antioxidant profile and better understand the hepatoprotective effects of basil phenolics, future research will assess additional markers, such as SOD, CAT, GSH, and GPx, alongside NRF2-regulated targets.

For mechanistic attribution, phenolics likely engage the Nrf2–KEAP1 antioxidant switch (release of Nrf2 via KEAP1 cysteine modification Cys151/Cys273/Cys288 or p62/SQSTM1-mediated sequestration [47]; induction of ARE targets: HO-1/HMOX1, NQO1, GCLC/GCLM, TXNRD1, SOD2/GPX with restoration of GSH pools) and temper NF-κB signaling (attenuation of IKKβ activity, stabilization of IκBα, reduced p65/RelA nuclear translocation and SIRT1-dependent deacetylation at K310; consequent downshift of TNF-α/IL-1β/IL-6, COX-2, and iNOS) [47,48,49,50,51,54], thereby aligning antioxidant, metabolic, and inflammatory axes with AMPK cross-talk (ACC phosphorylation → ↓malonyl-CoA → ↑CPT1A-driven β-oxidation [50,52]) and HO-1–derived CO/biliverdin–bilirubin, adding a further cytoprotective/anti-inflammatory tone [46,47].

Overall, basil phenolics appear to reset hepatic lipid economics: enhanced uptake and safe storage (lipid-droplet biogenesis) unload the bloodstream; a balanced rise in turnover (β-oxidation supplying ATP; lipogenesis re-esterifying fatty acids into inert droplets) prevents lipotoxic spillover; and the antioxidant environment suppresses metal-catalyzed initiation and preserves mitochondrial competence [11,12,16,28,29,30,35,36,37,38,46,47,48,49,50,51]. The enzyme signature is best read as transient membrane permeability under high flux rather than necrosis [56,57], and the lipoprotein picture (in a mouse framework) is directionally consistent with reduced atherogenic pressure when combined with improved redox status [58,59]. The preservation of lobular architecture, together with tempered shifts in ALT, ALP, and bilirubin, suggests an adaptive remodeling of hepatocytes under enhanced lipid flux, rather than cytotoxicity, consistent with controlled oxidative stress and maintained hepatic function. This constitutes a coherent adaptive phenotype: defense by metabolic routing plus redox governance, not by simple suppression of lipid supply [60].

Several studies have reported that supplementation with polyphenol-rich plant extracts can favorably modulate lipid and enzymatic profiles in animal models of hyperlipidemia. For instance, grape seed and green tea extracts were shown to decrease plasma triglycerides and LDL-C while maintaining or elevating HDL-C, accompanied by reductions in hepatic lipid peroxidation and ALT/AST activities [61,62,63,64]. Similarly, rosemary extract demonstrated significant hepatoprotective effects, including the normalization of serum transaminases and attenuation of oxidative stress markers in the liver [63,65]. Our findings with basil-enriched oil (OCBD) are consistent with these reports. We observed a redistribution of lipid fluxes toward the liver, a lower LDL/HDL ratio, and a predominance of ALT elevation within physiological limits, indicating adaptive hepatic remodeling rather than cytotoxicity. These parallels reinforce the notion that phenolic-rich extracts, including those from Ocimum basilicum, can simultaneously improve systemic lipid handling and maintain hepatic enzymatic homeostasis under conditions of dietary hyperlipidemia.

The dataset would benefit from (a) flux-resolved lipidomics and stable-isotope tracing to separate uptake, synthesis, oxidation, and export; (b) oxLDL and particle subfraction assays to refine cardiovascular interpretation; (c) molecular readouts along PPARα/PGC-1α (β-oxidation), SREBP-1c/ACC/FAS (lipogenesis), MTP/ApoB (VLDL export), SR-B1/LDLR (uptake), and Nrf2 targets to anchor the mechanistic model; (d) seahorse respirometry or high-resolution respirometry for mitochondrial function, and lipophagy/autophagy markers to test whether lipid clearance leverages lysosomal pathways; and (e) standardized NAFLD Activity Score with 4-HNE or MDA–protein adduct immunohistochemistry to link redox to histo-morphology.

To mechanistically interpret our bioassay readouts and identify which phytochemicals are most plausibly responsible, we performed structure-based molecular docking of the major phenolics caftaric, caffeic, chicoric, gallic, chlorogenic, and rosmarinic acids against six complementary targets spanning antioxidant defense, lipid oxidation, metabolic control, and cellular stress signaling. The KEAP1 Kelch domain (PDB 6TYM) is the proximal regulator of the NRF2 axis that drives antioxidant gene induction; PPAR-α (6KBA) and FXR (6A60) are nuclear receptors whose activities relate to lipid homeostasis and anti-inflammatory tone, aligning with radical-scavenging and inflammation modulation assays [23,48,49]. SIRT1 (catalytic core, 4I5I) represents a redox- and stress-responsive deacetylase contributing to cytoprotective pathways, while human 15-lipoxygenase-2/ALOX15B (7LAF) captures the chemistry around the iron-dependent active site that initiates lipid peroxidation, directly intersecting with membrane protection and hydroperoxide formation tests [50]. Finally, heterotrimeric AMPK (8BIK) embodies the principal cellular energy sensor linking metabolic signaling to anti-stress outcomes evident in our measurement panel. This structural framework, aligning functionally defined binding pockets with chemotypes enriched in catechol and carboxylate groups, provides an interpretive map that projects the endpoints of our measurements (radical quenching, suppression of lipid oxidation, and anti-inflammatory indicators) onto their molecular origins at the target level, enabling a rigorous discussion of plausible binding pathways.

Taken together, the docking landscape in Table 2 supports a poly-target mechanism dominated by rosmarinic and chicoric acids that maps directly onto the experimental endpoints, namely strong radical quenching, suppression of lipid autoxidation, and attenuation of pro-inflammatory signals, with the caveat that docking scores are semi-quantitative and were interpreted in the context of convergent bioassay evidence rather than as absolute affinities.

Before proceeding to residue-level interpretation, we note that chicoric acid produced the most favorable docking energy against SIRT1 (PDB 4I5I; −10.5 kcal mol^−1^; Table 2). This standout value, together with the ligand’s catechol–carboxylate chemotype, led us to prioritize the SIRT1–chicoric acid complex for detailed analysis to rationalize the observed high-affinity pose.

Prior to discussing residue-specific contacts, note that chicoric acid delivered the best docking energy versus SIRT1 (PDB 4I5I; −10.5 kcal·mol^−1^; Table 2). The combination of this exceptional score and the catechol–carboxylate scaffold led us to focus on the SIRT1–chicoric acid complex to explain its high-affinity binding mode. As shown in Figure 7, a PyMOL 3D view depicts the binding pocket (green) and the ligand (red). The left global surface view situates the pocket within the protein, whereas the right zoom reveals a deep, well-matched cavity with clear shape complementarity [66]. Such an enclosure enables a distributed stabilizing network of catechol/carboxylate-mediated hydrogen bonds, hydrophobic packing, and potential π–π interactions consistent with the favorable docking energy.

As shown in Figure 8, a dense hydrogen-bonding network surrounds chicoric acid. Eight H-bonds donated/accepted by carbonyl and phenolic OH groups effectively clamp both caffeoyl arms deep in the SIRT1 pocket, restricting torsional freedom and stabilizing the pose. Two further polar contacts, a C-H···O interaction to Glu261 and a π-donor hydrogen bond with Gln345, secure the placement and pre-organize the catechol rings for optimal shape complementarity [67]. Additional stabilization is provided by hydrophobic packing. Three π–alkyl contacts with Ile316, Ile347, and Ala262 seat the aromatic system against aliphatic side chains and reduce desolvation costs; a T-shaped π–π contact with Phe273 then locks one caffeoyl ring in a cross-stacked geometry [67,68]. Together, this ensemble of interactions explains the very favorable docking energy for the SIRT1–chicoric acid complex.

When docking data are integrated with the bioassay results, they support a synergistic, multi-target mechanism dominated by caffeoyl diesters, chiefly chicoric and rosmarinic acids. The emerging pattern centers on oxidative and lipid-stress axes: putative KEAP1-mediated NRF2 activation, SIRT1-linked cytoprotection, and ALOX15B-associated restraint of lipid peroxidation, with additional contributions from PPAR-α, FXR, and AMPK providing added support for metabolic homeostasis and attenuated inflammatory signaling. Chlorogenic and caftaric acids play secondary roles, while gallic acid shows limited involvement, reinforcing a synergistic multi-target mechanism rather than a single-target explanation.

ADMET profiling (Absorption, Distribution, Metabolism, Excretion, and Toxicity) is essential to judge whether the major phenolic constituents of the basil-enriched oil have a pharmacokinetic and safety profile compatible with long-term dietary use. In our context, ADMET modelling provides a translational bridge between the in vivo efficacy data and the molecular docking results, by indicating whether the most abundant phenolic acids behave like “acceptable” drug-like or nutraceutical-like molecules when compared to approved reference drugs (Figure 9, Table 3 [69,70,71]. Together, the ADMET-AI and ProTox-III data therefore support the view that the major phenolic constituents of the basil-enriched oil have a pharmacokinetic and safety profile that is broadly compatible with their proposed use as functional bioactives in a nutraceutical context.

Collectively, these results demonstrate that basil phenolics improve systemic lipid handling, preserve hepatic redox balance, and mitigate metabolic stress, supporting their potential as functional dietary supplements in hyperlipidemia.

## 5. Conclusions

This study demonstrates the significant potential of the basil-enriched oil as a natural hypolipidemic and hepatoprotective agent. Following a 12-week dietary intervention in mice, it significantly reduced plasma total cholesterol (TC), triglycerides (TG), and glucose levels, coupled with a concurrent increase in hepatic lipid content. This redistribution of lipids improved the LDL-C/HDL-C ratio and provided effective protection against diet-induced hypercholesterolemia, hepatosteatosis, and liver tissue damage.

These benefits, likely mediated through a combination of enhanced antioxidant defense and modulated hepatic lipid metabolism, underscore the extract’s potential as a valuable nutraceutical candidate for managing metabolic disorders. Future research should prioritize optimizing large-scale extraction, evaluating long-term safety, and further elucidating the precise molecular mechanisms, potentially involving the Nrf2 and PPARα pathways.

## Figures and Tables

**Table 1:** NAFLD Activity Score (NAS) for steatosis, lobular inflammation, and hepatocellular ballooning in liver sections from mice fed ND, OD, OCD, and OCBD diets.

Group	Steatosis (0–3)	Lobular Inflammation (0–3)	Hepatocellular Ballooning (0–2)	NAS Total (0–8)
**ND**	0	0	0	0
**OD**	1–2	1	0–1	2–4
**OCD**	2–3	2	1–2	5–7
**OCBD**	1	0–1	0–1	1–3

**Table 2:** Docking scores (kcal/mol) of basil phenolic acids against ALOX15B (7LAF), AMPK (8BIK), FXR (6A60), KEAP1 (6TYM), PPAR-α (6KBA), and SIRT1 (4I5I).

	Docking Score (Kcal/mol)					
Molecules	7LAF	8BIK	6A60	6TYM	6KBA	4I5I
Caftaric acid	−7.3	−7.2	−7.6	−8.3	−7.2	−9.2
Caffeic acid	−6.5	−6.1	−6.5	−7.1	−6.4	−6.8
Chicoric acid	−7.5	−7.4	−9.4	−9.7	−6.7	−10.5
Rosmarinic acid	−8.5	−7.7	−8.7	−9.5	−8.5	−9.3
Gallic acid	−5.6	−5.2	−5.7	−6.9	−6.1	−6.2
Chlorogenic acid	−7.3	−6.5	−8.0	−9.3	−8.4	−8.1

**Table 3:** ProTox-III in silico toxicity profile of the six major phenolic acids (1, caftaric; 2, caffeic; 3, chicoric; 4, rosmarinic; 5, gallic; 6, chlorogenic), including predicted LD_50_ values, toxicity class, and probabilities for hepatotoxicity, cardiotoxicity, carcinogenicity, mutagenicity, and cytotoxicity.

Molecules	Caftaric Acid	Caffeic Acid	Chicoric Acid	Rosmarinic Acid	Gallic Acid	Chlorogenic Acid
**N^o^**	1	2	3	4	5	6
**LD_50_ (mg/Kg)**	3800	2980	5000	5000	2000	5000
**Class**	5	5	5	5	4	5
Hepatotoxicity	Inactive	Inactive	Inactive	Inactive	Inactive	Inactive
Probability (%)	60	57	58	62	61	72
Cardiotoxicity	Inactive	Inactive	Inactive	Inactive	Inactive	Inactive
Probability (%)	92	97	68	69	89	99
Carcinogenicity	Inactive	Active	Inactive	Inactive	Active	Inactive
Probability (%)	54	78	51	66	56	68
Mutagenicity	Inactive	Inactive	Inactive	Inactive	Inactive	Inactive
Probability (%)	94	98	85	85	94	93
Cytotoxicity	Inactive	Inactive	Inactive	Inactive	Inactive	Inactive
Probability (%)	90	86	92	90	91	80

**Table 4:** Antioxidant activity of the BE.

Extract	ABTS Scavenging IC_50_ (µg/mL)	Linoleic Acid Oxidation IC_50_ (µg/mL)	Ferrous Ion Chelation IC_50_ (µg/mL)	Copper Ion Chelation IC_50_ (mg/mL)
BE	17.65 ± 0.1	111.31 ± 0.01	153.8 ± 2.5	0.544 ± 0.009

BE: Basil extract.

## Data Availability

The original contributions presented in this study are included in the article. Further inquiries can be directed to the corresponding author(s).

## Abbreviations

The following abbreviations are used in this manuscript:

ACC	Acetyl-CoA carboxylase
ACSL1/5	Acyl-CoA synthetase long chain 1/5
ALP	Alkaline phosphatase
ALT	Alanine aminotransferase
AMPK	AMP-activated protein kinase
ApoB	Apolipoprotein B
AST	Aspartate aminotransferase
BE	Basil extract
BEO	Basil-enriched oil
ChREBP	Carbohydrate-responsive element-binding protein
CPT1A	Carnitine palmitoyltransferase 1A
DGAT1/2	Diacylglycerol O-acyltransferase
FAT/CD36	Fatty acid translocase/CD36
FATP5	Fatty acid transport protein 5 (gene: SLC27A5)
FXR	Farnesoid X receptor
GGT	Gamma-glutamyl transferase
GPAT/AGPAT	Glycerol-3-phosphate/Acylglycerol-3-phosphate acyltransferases
HDL-C	High-density lipoprotein cholesterol
HO-1	Heme oxygenase-1
HPLC	High-performance liquid chromatography
IC_50_	Median inhibitory concentration
KEAP1	Kelch-like ECH-associated protein 1
LDL-C	Low-density lipoprotein cholesterol
LDLR	Low-density lipoprotein receptor
LIPC	Hepatic lipase
LPL	Lipoprotein lipase
MDA	Malondialdehyde
MTP	Microsomal triglyceride transfer protein
ND	Normal diet
NF-κB	Nuclear factor kappa-light-chain-enhancer of activated B cells
NLRP3	NOD-like receptor family pyrin domain-containing 3
NRF2	Nuclear factor erythroid 2–related factor 2
OCBD	Oil + cholesterol diet + basil extract
OCD	Oil + cholesterol diet
OD	Oil-enriched diet
PCSK9	Proprotein convertase subtilisin/kexin type 9
PGC-1α	Peroxisome proliferator-activated receptor gamma coactivator 1-alpha
PLIN2/5	Perilipin-2/5
PPAR-α	Peroxisome proliferator-activated receptor alpha
RSO	Refined soybean oil
SEM	Standard error of the mean
SHP	Small heterodimer partner
SIRT	Sirtuin 1
SR-B1	Scavenger receptor class B type 1
SREBP-1c	Sterol regulatory element-binding protein 1c
TC	Total cholesterol
TG	Triglycerides

## Appendix A

This table presents the nutritional information of refined soybean oil, as provided by the company producing it.

**Table A1:** Nutritional Composition of Refined Soybean Oil.

**Nutritional Information**	**Quantity per 100 g of Oil**
Saturated Fatty Acids	13.1 g
Monounsaturated Fatty Acids	28.4 g
Polyunsaturated Fatty Acids	58.3 g
Vitamin A	900 µg
Vitamin D3	7.5 µg

## Appendix B

The table represents the percentage of fat, protein, and carbohydrates, as well as the calculated energy content (kcal/g) for each diet. ND (normal diet), OD (RSO-supplemented diet), OCD (hypercholesterolemic diet), and OCBD (basil-enriched oil diet).

**Table A2:** Macronutrient and caloric composition of the four experimental diets.

Diet	Fat (%)	Protein (%)	Carbohydrate (%)	Energy (kcal/g)
ND (Normal diet)	4	20	60	3.56
OD (RSO-supplemented diet)	19.4	16.8	50.4	4.43
OCD (Hypercholesterolemic diet)	20.8	16.5	49.4	4.50
OCBD (Basil-enriched oil diet)	20.8	16.5	49.4	4.50

## Appendix C

Figure A1 illustrates the predicted ionization states and microspecies distribution of each ligand across a physiological pH range.
